# Unraveling the Physicochemical Properties and Bacterial Communities in Rabbit Meat during Chilled Storage

**DOI:** 10.3390/foods13040623

**Published:** 2024-02-19

**Authors:** Zhoulin Wu, Maoqin Xu, Wei He, Xiaoyu Li, Chaoqing Qiu, Jiamin Zhang

**Affiliations:** 1 Key Laboratory of Meat Processing of Sichuan, College of Food and Biological Engineering, Chengdu University, Chengdu 610106, China; wu9121@126.com (Z.W.); qinqiner688@163.com (M.X.); hwei032424@163.com (W.H.); lixiaoyu9918@163.com (X.L.); 18787336508@163.com (C.Q.); 2 Development and Research Center of Sichuan Cuisine, Sichuan Tourism University, Chengdu 610100, China

**Keywords:** rabbit meat, chilled storage, physicochemical properties, high throughput sequencing, spoilage

## Abstract

The freshness and bacterial communities of fresh and salted rabbit meat during 8 days of refrigerated storage at 4 °C were evaluated. The results showed that the addition of 2% salt significantly changed the color of meat, of which the lightness (L*), redness (a*), and yellowness (b*) were lower than that of fresh meat over time. The pH of all samples increased during storage, and meat with salt addition had lower values in comparison to fresh samples over time. The total volatile base nitrogen (TVB-N) concentration increased rapidly in salt-treated meat but was significantly (*p* < 0.05) lower than that in meat without salt added before 6 days. Over time, the content of thiobarbituric acid reactive substances (TBARS) showed a progressive trend, but a rapid increase occurred in salted meat. High-throughput sequencing showed that the microflora of each sample had a positive trend in alpha diversity and a negative trend in beta diversity. Bacterial taxonomic analysis indicated that the initial microbial flora for chilled rabbit meat was dominated by *Shigaella*, *Bacteroides*, and *Lactococcus*, and the population of *Brochothrix* and *Psychrobacter* increased over time and became the dominant spoilage bacterium. In particular, the addition of salt significantly reduced the abundance of *Psychrobacter* and *Brochothrix*. These findings might provide valuable information regarding the quality monitoring of rabbit meat during chilled storage.

## 1. Introduction

Rabbit meat offers excellent functionality and has better nutritional and dietary properties than red meat does due to its low contents of fat, cholesterol, and sodium and high contents of protein and essential amino acids [1,2]. Although rabbit meat is marketed and consumed worldwide, its consumption accounts for less than 3% of all meat consumed in the European Union [3]. China produces about 60% of the world’s total rabbit meat, and various types of meat products including primarily and processed products have been developed in recent years [4]. Nonetheless, rabbit meat is still mainly marketed as cut-up parts (such as hind legs and loins) or whole carcasses in China [4] and Italy [5]. Normally, fresh meat is one of the most prominent food categories for young ones, and its presentation is an important factor influencing a consumer’s preference and willingness to purchase; thus, more and more people are seeking ways to optimize the shelf life of fresh meat, but its relatively short life limits its potential value.

The high nutritious and moisture content in fresh meat makes it the most perishable food, with the colonization and development of a wide consortium of microorganisms [6]. There is an approximate annual loss of 20% of the initial meat production, with 60.84 million tons lost or wasted due to spoilage every year worldwide [7]. Spoilage is a complex process, and its manifestation is dictated by the nature of the meat itself, the meat species, animal husbandry, the hygiene levels of the process, and storage conditions [8]. It has been confirmed that the spoilage of fresh meat is mainly caused by bacteria, and species composition and taxa abundance have a major impact on spoilage status [8]. In recent years, the bacterial communities that precede meat spoilage have been widely examined through culture-independent methods [9,10,11,12], and amplicon-sequencing approaches targeting the 16S rRNA gene allowed us to achieve a better assessment of species richness and microbial communities, which has increased the resolution of meat spoilage-related microbiota analysis. However, linking a spoilage defect to community structure profiling is difficult since the bacterial community is very complex and dynamic. Furthermore, a diversity of characteristics has been enrolled to evaluate the spoilage defects of meat products, such as pH and texture changes, slime or liquid formation, off-odors, various color deteriorations, or structural components’ degradation, but these defects take place by different mechanisms [13]. The combination of physicochemical parameters and 16S rRNA sequencing is a powerful tool for exploring the relationship between microorganism communities and meat quality [14]. Up to now, information on the correlation between these physicochemical parameters and bacterial communities in fresh rabbit meat has been very scarce.

Storage temperature is closely related to the shelf life and quality of fresh meat. Generally, chilled (0 to 4 °C) and frozen (−18 to −40 °C) are the most two common commercial storage conditions, of which, chilled storage is critical for meat’s hygiene, appearance, safety, and nutritional quality [15,16]. However, meat with chilled storage has a limited shelf life, primarily due to microbial growth and endogenous proteolytic enzyme activities [6]. The type of microorganisms and their loads depend on the specific meat and meat products, which can finally affect the rate of the spoilage process. It has been shown that poultry meat is spoiled quickly, but the dominant spoilage genera are quite variable among fresh meat types such as poultry [17], pork [18], lamb [19], and beef [20] during chilled storage. However, it is remains largely unknown about the predominant microflora of rabbit meat during chilled storage. In this study, both fresh and salted rabbit meat were stored at 4 °C for eight days, during which, the freshness and bacteria communities were analyzed. Our results may provide guidelines regarding the optimum method of chilled storage for rabbit meat depending on basic data of the physicochemical indicators and 16S rRNA sequencing. The results of the first part of this study will be presented, while the main spoilage-related bacterial genera and metabolites as well as their interaction network differences among different species of meat such as poultry, pork, and yak meat will be presented in the subsequent companion research.

## 2. Materials and Methods

### 2.1. Meat Sample Collection and Storage

This study was conducted with a batch of commercial Hyla meat rabbits at a local farm. All rabbits were reared under similar management conditions, and detailed nutritional information was described in our earlier report [21]. Animals were sold at 70 days of age and transported to a local abattoir (Chengdu, China). A total of 30 rabbits with an average body weight of 2148.27 ± 95.22 g were randomly selected and then subjected to electrical stunning followed by exsanguination, skinning, and evisceration procedures. After the slaughtering, relatively homogenous portions of left hind legs were collected and transported to the laboratory within 2 h, followed by being aseptically cut into pieces of uniform shape and size, weighing 40.94 ± 1.37 g each with thicknesses of 3.7 ± 0.4 cm. Meat pieces with (Group A, N = 15) or without 2% salt (Group B, N = 15) were packed in trays and wrapped with polyvinylchloride films and then stored at 4 °C for 0–8 days. Moreover, meat pieces were removed from films on each analysis day for physicochemical determination and microbiota identification.

### 2.2. Physicochemical Analysis

The pH value, color, and thiobarbituric acid reactive substances (TBARS) of rabbit meat samples were determined according to our previous report [22]. Briefly, the pH value of all samples was evaluated using an insert electrode pH-star (Matthäus, Pöttmes, Germany). The color of rabbit meat samples covered indicators of L* (lightness), a* (redness), and b* (yellowness), which were measured by using a color measurement device (Minolta, CR-300). The TBARS tests were determined as described previously [23], and the values were expressed as mg of malondialdehyde (MDA) per kg of sample. Referring to the national standard (GB 5009.228-2016), the TVB-N (total volatile basic nitrogen) concentration in samples was measured according to the semi micro nitrogen determination method. Briefly, in 10 g of meat, the connective tissue was removed, and fatty deposits were homogenized in 100 mL of distilled water, and the mixture was equilibrated at room temperature for 30 min, shaking every 10 min by a thermostat magnetic stirrer. A 10 mL filtrate aliquot was added to 10 mL of magnesium oxide followed by distillation lasting for 5 min, and steam distillation was carried out. Then, the TVB-N was calculated by the consumption of 0.1 mol/L hydrochloric acid solution, and finally, it was expressed as mg/100 g fresh sample.

### 2.3. DNA Extraction and Sequencing

Bacterial DNA was extracted with the QIAamp DNA Stool Mini Kit (Qiagen, Shanghai, China) as per the manufacturer’s instructions. A NanoDrop ND-1000 spectrophotometer (NanoDrop Technologies, Montchanin, DE, USA) was used to measure the DNA concentration, while the purity was checked by gel electrophoresis. Thereafter, amplicons targeting the V3–V4 hypervariable region of 16S rRNA genes were generated by PCR using a range of universal primers (338F and 806R) with barcode sequences. The cycling condition including initial denaturation (95 °C, 3 min), 25 cycles of denaturation (95 °C, 30 s), annealing (55 °C, 30 s), elongation (30 s, 72 °C), and final extension for 10 min at 72 °C was performed for the PCR reactions. Each sample was amplified in triplicate, and the products were pooled and purified using the an EasyPure PCR Purification Kit (TransGen, Beijing, China). A DNA library was constructed using an Illumina TruSeq kit (Illumina, San Diego, CA, USA) following the manufacturer’s specifications, and sequencing of the libraries was conducted on the Illumina HiSeq 2500 platform for the generation of 2 × 250 bp paired-end reads.

### 2.4. Bioinformatics and Data Analysis

The raw sequences were assigned to samples based on their unique barcode, and the Trimmomatic (v0.33) [24] and fastp software (v0.19.8) [25] were used to screen the qualified raw reads. Cutadapt software (1.9.1) [26] was enrolled to identify and trim the adaptor sequences for obtaining high-quality target reads. Subsequent bioinformatics analysis was carried out using the open source software Qiime2 (v2023.2) [27] for merging paired-end reads, demultiplexing, and picking de novo operational taxonomic units (OTUs). Representative OTU sequences were aligned using the DEBLUR program [28] integrated within QIIME2. A naïve Bayesian classifier was trained on the Silva v138 database and further used for the classification of these OTUs into specific taxa. The richness (observed features) and diversity (Shannon) indices of the microbial composition were analyzed using QIIME2 by the Kruskal–Wallis test. Beta diversity was calculated through the Bray–Curtis and Weighted Unifrac metrics using q2-diversity in QIIME2; thereafter, dissimilarities were visualized through the principal coordinates analysis (PCoA) method generated with ggplot2 (v3.3.6) from R package [29]. Statistical analyses were performed using R (v4.1.3) software (https://www.r-project.org/, (accessed on 10 March 2022)). The criterion of significance was conducted at *p* < 0.05 and the values were presented as the means.

## 3. Results

### 3.1. Physicochemical Changes of Rabbit Meat

**Figure 1:**
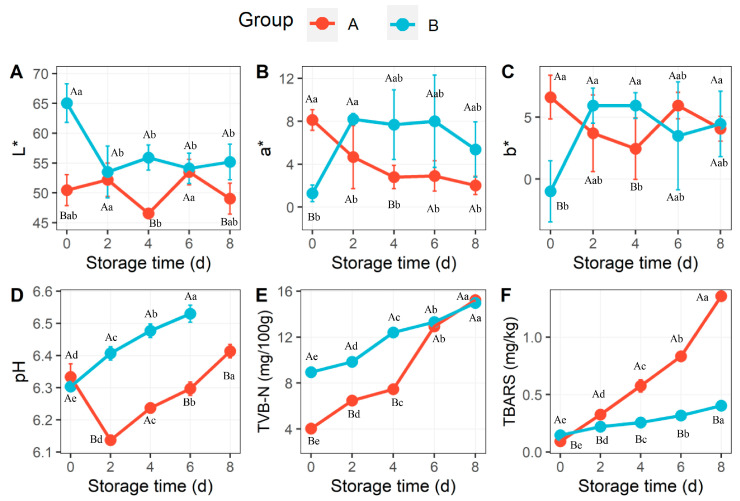
The changes in L* (**A**), a* (**B**), b* (**C**), pH (**D**), TVB-N (**E**), and TBARS (**F**) in rabbit meat during storage at 4 °C. The different lowercase letters (a–c) indicate significant differences across the storage times (0 d, 2 d, 4 d, 6 d, 8 d) within groups, while different capital letters (A, B) indicate significant differences (*p* < 0.05) between meat groups, respectively. Error bars derived from the standard deviation of replicates.

The changes in color, pH, thiobarbituric acid reactive substances (TBARS), and total volatile basic nitrogen (TVB-N) concentration in the A and B groups are shown in Figure 1. The lightness (L*) showed a significant change due to salt treatment at the beginning of storage, and the L* value was always higher in group B over time (Figure 1A). Both redness (a*) and yellowness (b*) were significantly higher in group A than in group B at the beginning of storage, but they exhibited lower values in group A during storage (Figure 1B,C). The pH values of rabbit meat from two groups were similar at the beginning of storage and fluctuated during storage, which varied from 6.12 to 6.61, as shown in Figure 1D. Furthermore, meat from group B had a significant higher pH value compared to that from group A in each storage stage. The TVB-N concentration increased rapidly in group A but was significantly lower than that in group B before 6 days, and a sharp increase was found after 4 days and 2 days in group B and group A, respectively. It should be noted that the value was similar in these groups after 6 days and reached up to 15.05 mg/kg (Figure 1E). The TBARS results showed a progressive trend in both groups but a rapid increase in group A, and they showed substantially higher values in group B compared with group A (Figure 1F).

### 3.2. Summary of Sequencing Data and Taxonomy

**Figure 2:**
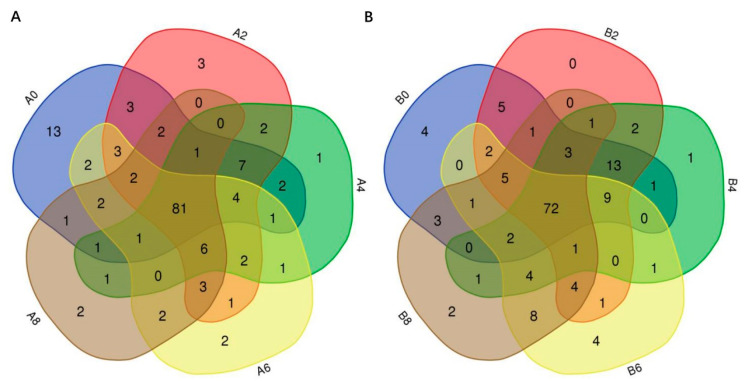
The unique and shared OTUs of the bacterial communities in rabbit meat from group A (**A**) and group B (**B**) during storage at 4 °C. (A0, A2, A4, A6, A8: meat pieces of group A at chilled storage for 0 d, 2 d, 4 d, 6 d, and 8 d, respectively; B0, B2, B4, B6, B8: meat pieces of group B at chilled storage for 0 d, 2 d, 4 d, 6 d, and 8 d, respectively).

High-throughput sequencing was employed to characterize the bacterial community composition and diversity in the rabbit meat samples. Following the screening pipeline, a total of 2,351,329 sequences with an average of 78,378 per sample were ultimately retained after quality control processing. The results from rarefaction curves showed that our sequencing depth had met the analysis requirements, because the sequencing depth reached the maximum, as the rarefaction curves showed a saturated tendency when the sequence number was nearly 5000 (Figure S1). Using the DEBLUR program in QIIME2, all the clean sequences were assigned to 927 OTUs. Regarding the OTU abundance matrix, a Venn diagram was constructed to show the number of OTUs common to each group among storage stages. As shown in Figure 2A, a total of 81 common OTUs were found in rabbit meat from group A among different storage periods, and most of the unique OTUs, of which there were 13, were observed at the beginning of storage (Figure 2A). On the other hand, a total of 72 common OTUs were found in rabbit meat from group B among different storage periods, and most of the unique OTUs, of which there were four, were observed at the beginning of storage and 6 days after storage (Figure 2B).

### 3.3. The Diversity of Bacterial Communities

**Figure 3:**
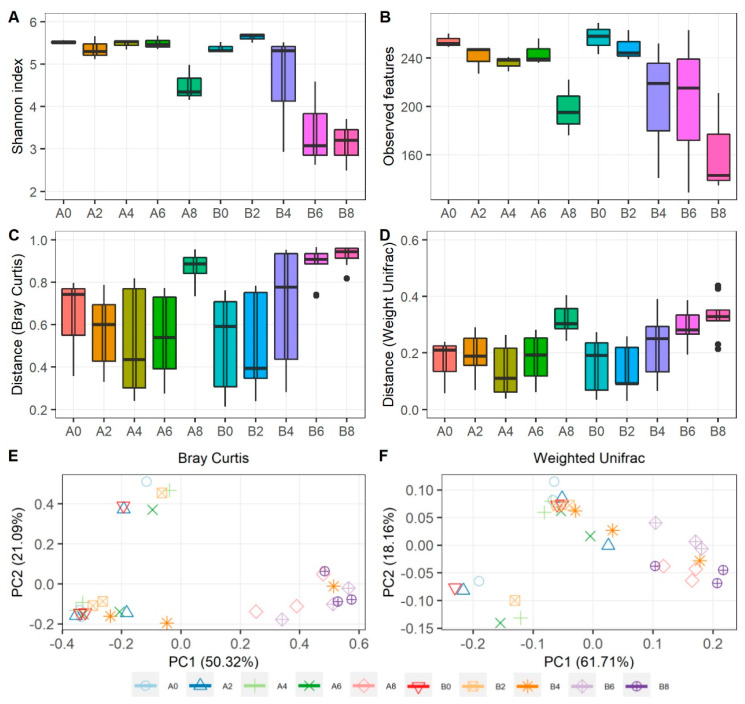
The bacterial community diversities of rabbit meat from group A and group B during storage at 4 °C. The species diversity of Shannon (**A**) and species richness of observed features (**B**). Boxplot showed the distances from Bray–Curtis distance matrices (**C**) and Weighted UniFrac distance matrices (**D**). The bacterial community structure shown by principal coordinate analysis (PCoA) of Bray–Curtis (**E**) and Weighted UniFrac distances (**F**). (A0, A2, A4, A6, A8: meat pieces of group A at chilled storage for 0 d, 2 d, 4 d, 6 d, and 8 d, respectively; B0, B2, B4, B6, B8: meat pieces of group B at chilled storage for 0 d, 2 d, 4 d, 6 d, and 8 d, respectively).

To further assess the community changes in rabbit meat in both groups over time, the species diversity (Shannon) and richness (observed features) were analyzed. As shown in Figure 3A,B, both the Shannon and observed features indexes declined noticeably in group B during storage. However, only a marked decline was found after 6 days of storage in group A. Then, Bray–Curtis and Weighted Unifrac were used to calculate the beta diversity distance matrices. Although all changes were not statistically significant, increases occurred for both groups throughout storage (Figure 3C,D). Furthermore, comparisons of the bacterial community structure of meat samples were performed using the Bray–Curtis and Weighted Unifrac metrics from which distance matrices were constructed and visualized using PCoA (Figure 3E,F). Samples from each storage period tended to be clustered together, but there was the less-defined clustering of salted versus un-salted meat samples.

### 3.4. The Composition of Bacterial Communities

**Figure 4:**
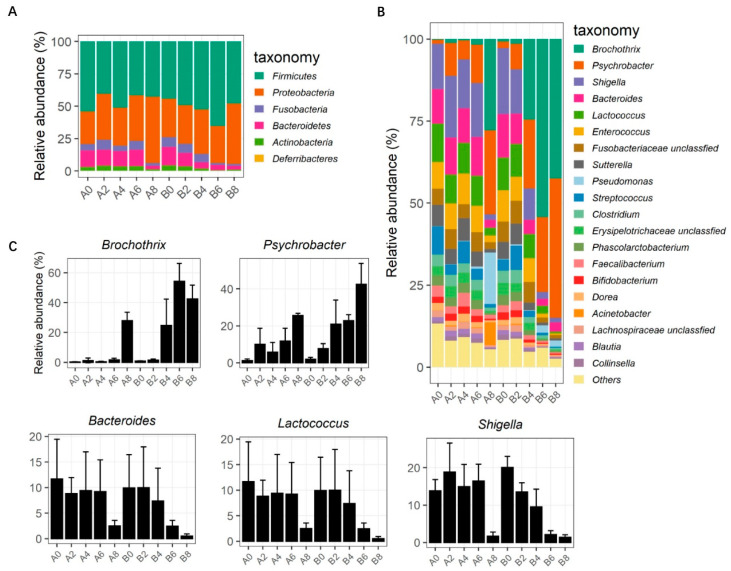
Relative abundance of bacteria at the phylum (**A**) and genus (**B**) levels (top 20), and changes in relative abundance of each bacterial genera during storage (**C**). (A0, A2, A4, A6, A8: meat pieces of group A at chilled storage for 0 d, 2 d, 4 d, 6 d, and 8 d, respectively; B0, B2, B4, B6, B8: meat pieces of group B at chilled storage for 0 d, 2 d, 4 d, 6 d, and 8 d, respectively).

The relative proportions of preponderant taxa at the levels of phylum and genus were calculated in all samples. At the phylum level, *Firmicutes* was still the main bacterium for both groups during the storage period. *Firmicutes* and *Proteobacteria* were the major bacteria that together represented >83% of the sequences (Figure 4A). At the genus level, a total of 107 genera were identified, and the top 20 genera are shown in Figure 4B. The composition of bacterial communities in the samples underwent significant fluctuations between groups and among storage periods. On day0, the dominant genera were *Shigella*, *Bacteroides*, and *Lactococcus* in both groups, of which the proportion of *Shigella* and *Bacteroides* decreased significantly in meat from group B during the storage period. On the other hand, the proportion of *Psychrobacter* and *Brochothrix* increased obviously in all meat samples during storage, and *Brochothrix* was particularly abundant in meat samples from group B (Figure 4C).

## 4. Discussion

Traditional methods of meat presentation mainly include drying, brining, fermentation, refrigeration, and canning; these methods are aimed to minimize oxidation and enzymatic spoilage and inhibit microbial spoilage [16]. However, new presentation methods are broadly categorized as controlling temperature, controlling water activity, and the use of chemical or biopreservatives [13,16]. The addition of salt is a widely used chemical method of meat presentation, but we know very little about characteristics of rabbit meat with salt treatment. Appearance is the major criterion for purchase selection and the initial evaluation of fresh meat quality [30]. It is well known that meat color is a critical food quality attribute. Generally, a bright red color denotes freshness [31]. In this study, we found that meat treated with 2% salt had relatively lower lightness as well as lower redness and yellowness. Similar results have been reported for goat meat [32] at 4 °C storage, for which the meat discoloration possibly caused by the pro-oxidative activity of salt and released iron from heme pigments and other heme-binding molecules. According to previous reports, the decline in a* value can be caused by oxidation and the formation of metmyoglobin, while the b* value is associated with yellow pigment formed from reactions between lipids and amines during storage [31,33]. pH is another important indicator for meat freshness since it reflects the degree of protein degradation [34]. The final pH of meat was influenced by multiple factors such as pre-slaughter practices, the rigidity process post-slaughter, and preservation conditions, but fresh meat begins to deteriorate at around pH 6.3–6.5 [35]. In the current study, the pH of all meat samples increased during storage due to the production of organic acids by bacteria in the anaerobic metabolism. Mechanistically, pH changes as a result of the processes of microbial growth releasing carbon dioxide [36]. This finding was similar to a study conducted using fresh turkey sausages [37]. In addition, rabbit meat from group A had lower pH values than that of meat from group B, which might be due to the fact that the salt treatment inhibited microbial growth since a higher pH in meat favors bacteria growth [17]; in particular, Gram-negative bacteria cause an increase in ammoniacal nitrogen levels and a degradation of proteins and amino acids [38]. Therefore, the addition of salt in rabbit meat suppressed the growth of bacteria but decreased lightness, redness, and yellowness. As for rabbit meat, appearance and texture are of utmost importance to consumers; therefore, attention may be paid with regard to storage conditions to preserve acceptability. For example, modified-atmosphere packages [2] as well as plant extracts [39] are effective in preserving physical or sensory traits and microbial statuses, which implies that these areas contribute to improving the storage quality of rabbit meat.

TVB-N is an important quality indicator of fresh meat [40], which refers to the decomposition of protein in animal food due to the action of enzymes and microorganisms to produce ammonia, amines, trimethylamine and other basic nitrogen-containing substances [6]. The TVB-N concentrations increased rapidly before day 6 in all samples but were significantly slower in group A than those in group B (Figure 1E), indicating that the addition of salt resulted in the slower decomposition of protein. On day 8, the TVB-N concentration in both groups exceeded the spoilage limits stipulated by Chinese National Food Safety Standard GB2707-2016 (≤15 mg/kg). Similar results have been reported, in which TVB-N was positively correlated with the storage period, and which strongly indicates continuous deterioration during storage [41,42]. The lipid oxidization of meat was calculated by measuring thiobarbituric acid reactive substances during storage, from which we found a constant increase but significant difference in TBARSs during storage between groups. Meat with salt treatment showed higher (*p* < 0.05) and rapidly increased TBARS values, which indicated a strong oxidative effect of salt in rabbit meat. According to the literature, the addition of salt to meat and meat products resulted in promoting lipid oxidation [32].

Spoilage microorganisms could originate from animal microbiota, production plants, or human manipulators in the production chain [43]. The type of microorganisms and their loads depend on the specific meat and meat products, which can finally affect the rate of the spoilage process. It has been showed that the commonest microbial genera isolated from fresh meat are *Acinetobacter*, *Pseudomonas*, *Brochothrix*, *Flavobacterium*, *Psychrobacter*, *Moraxella*, *Staphylococcus*, *Micrococcus*, lactic acid bacteria, and *Enterobacteriaceae* [44]. Poultry meat is spoiled quickly, and the specific bacteria include *Pseudomonas*, *Enterobacteriaceae*, lactic acid bacteria, and *Brochothrix thermosphacta* [17]. Lamb meat becomes spoiled by *Pseudomonas*, followed by *Brochothrix Ralstonia*, *Psychrobacter,* and *Acinetobacter* for chilling and supercooling storage [19], whereas *Leuconostoc*, *Lactobacillus*, *Pseudomonas taetrolens,* and *Pseudomonas fragi* are the typical spoilage bacteria for beef [20]. For pork, *Pseudomonas*, *Acinetobacter*, *Pantoea*, *Brochothrix,* and *Raoultella* are the dominant genera for chilling storage [18]. *Pseudomonas* and *Brochothrix* accounted for almost 90% of the identified spoiled genera [10], and these genera were reported as predominant bacteria in the aerobic packing of raw meat [45]. Unlike these types of meat mentioned above, the initial microbial flora for chilled rabbit meat was dominated by *Shigella*, *Bacteroides*, and *Lactococcus*, and the population of *Brochothrix* and *Psychrobacter* was increased over time and reached over half of total population, particularly after 6 days of storage (Figure 4C). This finding adds to the current knowledge, indicating that *Brochothrix* species dominate the bacterial communities after 7 days of aerobic storage for beef [46], which is a dominant spoilage organism that plays a role in shortening the self life of aerobically stored meat [47]. Furthermore, we found that *Psychrobacter* was another dominant bacterial taxa among the storage flora of chilled rabbit meat, and earlier studies have investigated its incidence in chilled rabbit meat [48]. However, the relationship between *Psychrobacter* and chilled rabbit meat spoilage remains poorly understood and deserves further investigation.

The goal of this study was to compare the freshness and bacteria community differences in fresh and salted meat to lay a foundation for prolonging chilled rabbit meat’s shelf life. However, our work has several limitations. As it enrolled relatively homogenous portions of left hind legs, the published work shows that the chemical composition and quality of rabbit meat varies among cuts [49] and that metabolic activity differences can be noticeable, which therefore might contribute to the variation in results when focusing on other carcass parts. However, there is lack of publications showing that during chilled storage, different carcass cuts differ in spoilage microbiota. Further study would have to address this question. Here, we also compared the physicochemical parameters for meat with or without salt, including color, pH, TVB-N, and TBARS measurements. Since, in our study, model calculations and validation experiments were not performed, inferred mechanisms may be limited.

## 5. Conclusions

This study assessed the physicochemical properties and bacterial communities of rabbit meat with or without added salt during refrigerated storage at 4 °C. The addition of salt could bring several characteristic modifications concerning both microbial load and appearances. The dominant genera of fresh rabbit meat were *Shigella*, *Bacteroides*, and *Lactococcus*, while *Psychrobacter* and *Brochothrix* were the main dominant spoilage bacteria, and the addition of salt significantly reduced the abundance of *Psychrobacter* and *Brochothrix*. As seen from the physicochemical parameters, meat treated with salt had relatively higher pH values but lower sensorial scores as well as slower increasing speeds and lower TVB-N and TBARS concentrations. This indicated that salt addition significantly slowed down the rate of decomposition of protein but promoted lipid oxidation and decreased the appearances of rabbit meat; thus, future works are needed to better the application of salt addition and especially improve the sensor properties.

## Data Availability

The original contributions presented in the study are included in the article/Supplementary Material, further inquiries can be directed to the corresponding author.

## Supplementary Materials

The following supporting information can be downloaded at: https://www.mdpi.com/article/10.3390/foods13040623/s1, Figure S1. Rarefaction curves of the meat microbiota. Curves of observed features (A), faith PD (B), and shannon indices (C).
