# Supplementary material for: Unraveling the Physicochemical Properties and Bacterial Communities in Rabbit Meat during Chilled Storage
Source: Foods. 2024 Feb 19;13(4):623. doi: 10.3390/foods13040623 (PMC10887707; doi:10.3390/foods13040623)
Supplement: Supplementary file 1 [file foods-13-00623-s001.zip › foods-2852910-supplementary.pdf]

1.1 Supplementary Figures

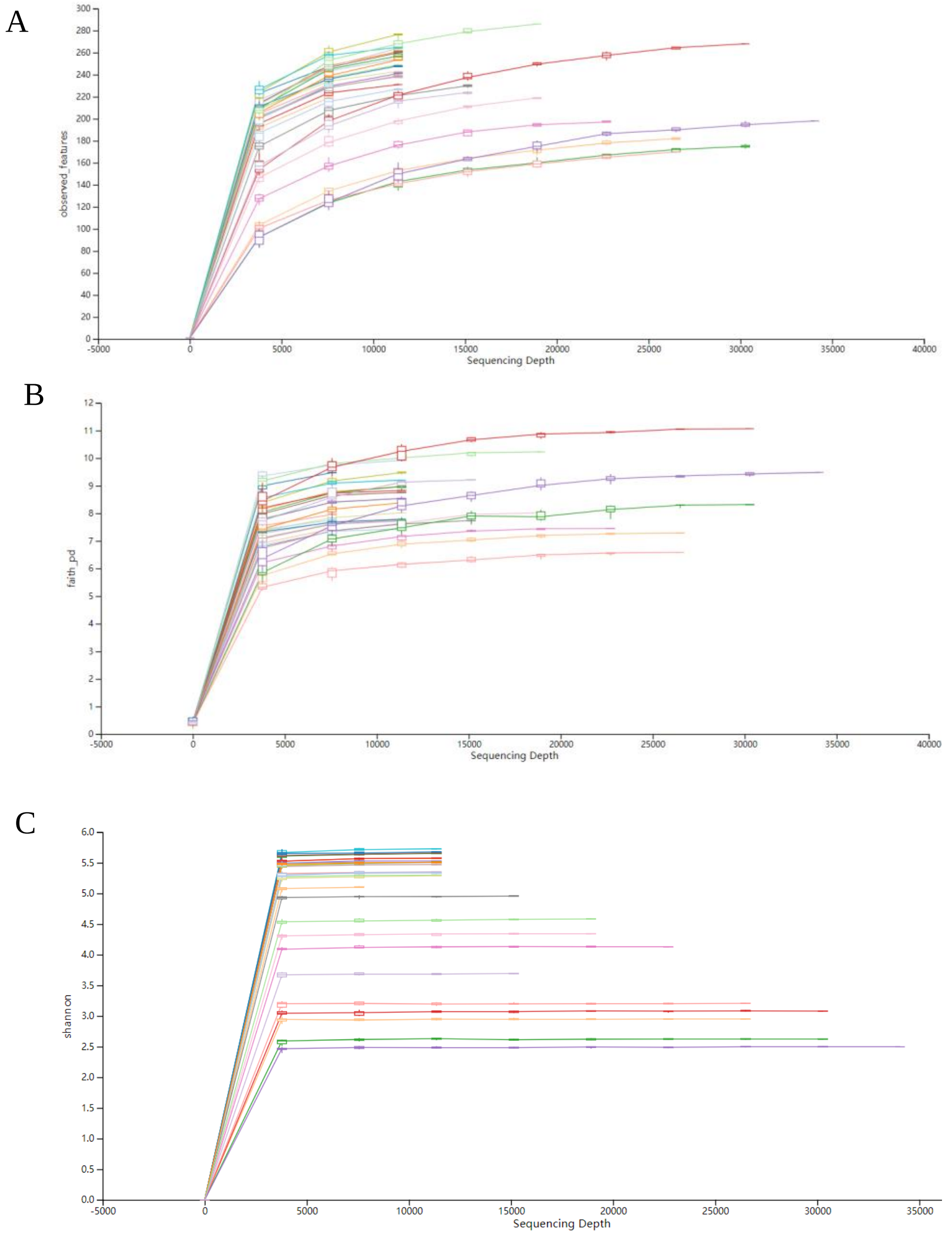

Figure S1. Rarefaction curves of the meat microbiota. Curves of observed features (A), faith PD (B), and shannon indices (C).
